# Methods to Discover and Validate Biofluid-Based Biomarkers in Neurodegenerative Dementias

**DOI:** 10.1016/j.mcpro.2023.100629

**Published:** 2023-08-07

**Authors:** Charlotte E. Teunissen, Leighann Kimble, Sherif Bayoumy, Katharina Bolsewig, Felicia Burtscher, Salomé Coppens, Shreyasee Das, Dea Gogishvili, Bárbara Fernandes Gomes, Nerea Gómez de San José, Ekaterina Mavrina, Francisco J. Meda, Pablo Mohaupt, Sára Mravinacová, Katharina Waury, Anna Lidia Wojdała, Sanne Abeln, Davide Chiasserini, Christophe Hirtz, Lorenzo Gaetani, Lisa Vermunt, Giovanni Bellomo, Steffen Halbgebauer, Sylvain Lehmann, Anna Månberg, Peter Nilsson, Markus Otto, Eugeen Vanmechelen, Inge M.W. Verberk, Eline Willemse, Henrik Zetterberg, MIRIADE consortium

**Affiliations:** 1 MIRIADE Consortium, Multiomics Interdisciplinary Research Integration to Address DEmentia diagnosis, Amsterdam, The Netherlands; 2 Neurochemistry Lab, Department of Laboratory Medicine, Amsterdam Neuroscience, Amsterdam UMC, Vrije Universiteit, Amsterdam, Netherlands; 3 KIN Center for Digital Innovation, Vrije Universiteit Amsterdam, Amsterdam, Netherlands; 4 Luxembourg Centre for Systems Biomedicine, University of Luxembourg, Esch-sur-Alzette, Luxembourg; 5 National Measurement Laboratory at LGC, Teddington, United Kingdom; 6 ADx NeuroSciences, Gent, Belgium; 7 Department of Computer Science, Vrije Universiteit Amsterdam, Amsterdam, Netherlands; 8 Department of Psychiatry and Neurochemistry, Institute of Neuroscience and Physiology, The Sahlgrenska Academy at the University of Gothenburg, Mölndal, Sweden; 9 Department of Neurology, University of Ulm, Ulm, Germany; 10 LBPC-PPC, IRMB CHU Montpellier, INM INSERM, Université de Montpellier, Montpellier, France; 11 Division of Affinity Proteomics, Department of Protein Science, KTH Royal Institute of Technology, SciLifeLab, Stockholm, Sweden; 12 Section of Neurology, Department of Medicine and Surgery, University of Perugia, Perugia, Italy; 13 Section of Physiology and Biochemistry, Department of Medicine and Surgery, University of Perugia, Perugia, Italy; 14 German Center for Neurodegenerative Diseases (DZNE e.V.), Ulm, Germany; 15 Department of Neurology, Martin-Luther-University Halle-Wittenberg, Halle, Germany; 16 Clinical Neurochemistry Laboratory, Sahlgrenska University Hospital, Mölndal, Sweden; 17 Department of Neurodegenerative Disease, UCL Institute of Neurology, London, UK; 18 UK Dementia Research Institute at UCL, London, UK; 19 Hong Kong Center for Neurodegenerative Diseases, Clear Water Bay, Hong Kong, China; 20 Wisconsin Alzheimer’s Disease Research Center, University of Wisconsin School of Medicine and Public Health, University of Wisconsin-Madison, Madison, Wisconsin, USA

**Keywords:** mass-spectrometry, immunoassays, protein detection, cerebrospinal fluid, blood, biomarkers, validation

## Abstract

Neurodegenerative dementias are progressive diseases that cause neuronal network breakdown in different brain regions often because of accumulation of misfolded proteins in the brain extracellular matrix, such as amyloids or inside neurons or other cell types of the brain. Several diagnostic protein biomarkers in body fluids are being used and implemented, such as for Alzheimer’s disease. However, there is still a lack of biomarkers for co-pathologies and other causes of dementia. Such biofluid-based biomarkers enable precision medicine approaches for diagnosis and treatment, allow to learn more about underlying disease processes, and facilitate the development of patient inclusion and evaluation tools in clinical trials. When designing studies to discover novel biofluid-based biomarkers, choice of technology is an important starting point. But there are so many technologies to choose among. To address this, we here review the technologies that are currently available in research settings and, in some cases, in clinical laboratory practice. This presents a form of lexicon on each technology addressing its use in research and clinics, its strengths and limitations, and a future perspective.

## Highlights

• Biomarkers in body fluids have clinical value in neurodegenerative dementias.
• There are several technologies for discovery and implementation of biomarkers.
• We review these technologies for their value in research and clinics.

## Graphical Abstract

**Figure:**
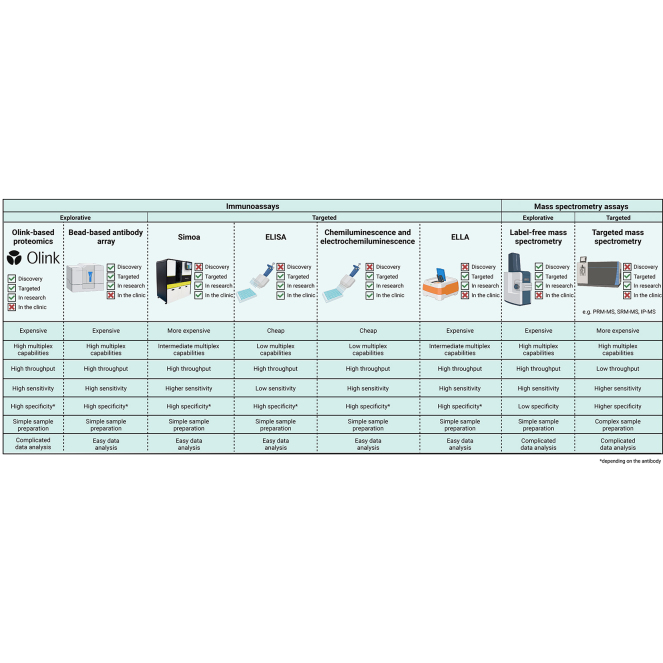

## In Brief

We review technologies used for body fluid protein biomarker discovery and translation to clinical practice. We address their main characteristics, their use in research and clinics, strengths and limitations, and a future perspective.

Neurodegenerative dementias are progressive diseases that cause neuronal network breakdown in different brain regions often because of accumulation of misfolded proteins in the brain extracellular matrix, such as amyloids or inside neurons or other cell types of the brain (1, 2). The abnormal protein accumulations may directly impair protein homeostasis and function of neurons. They may also cause astrocytic and microglial activation that may have both beneficial, for example, a protective response to remove the protein accumulations and rejuvenate the brain, or detrimental, for example, overactivation that may cause inflammation, oxidative stress, and energy crisis, effects on the brain. Synaptic dysfunction and neuronal network breakdown eventually cause clinical symptoms and dementia, when resilience and network redundancies and compensatory mechanisms have been exhausted; the precise nature of the clinical phenotype of the patient is determined by which brain regions are affected (3).

Alzheimer’s disease (AD)-related pathologies, key among which are extracellular amyloid β (Aβ) plaques, intraneuronal tau tangles, and neurodegeneration, are evident in the brain decades before symptom onset (4). It is increasingly recognized that a presymptomatic phase whereby pathologies accumulate years before symptoms is a common feature of most neurodegenerative dementias (5, 6). While reliable cerebrospinal fluid (CSF), blood, and imaging biomarkers for these AD pathologies have been available for some time (7) and promising data on CSF biomarkers for α-synuclein pathology exist (8), there is still a lack of reliable biomarkers for TAR DNA-binding protein (TDP-43) inclusions, a common pathology in some forms of frontotemporal dementia (FTD) and amyotrophic lateral sclerosis (ALS), which can be found in AD and other neurodegenerative dementias as well and other neurodegenerative changes that may involve particular aspects of neuronal, astrocytic and microglial dysfunction, blood-brain barrier dysfunction, myelin breakdown, and a host of other potentially disease-related processes.

Clearly, more biofluid-based biomarkers for neurodegenerative dementias are needed to enable precision medicine approaches for diagnosis and treatment, to learn more about underlying disease processes, and to facilitate the development of patient inclusion and evaluation tools in clinical trials. In the early phase of drug discovery projects, researchers are nowadays encouraged, both by funders and regulatory agencies, to develop a translatable biomarker pipeline of relevance to the drug target and the potential mechanism of action of the drug.

When designing studies to discover novel biofluid-based biomarkers, choice of technology is an important starting point. But there are so many technologies to choose among. To address this, the MIRIADE consortium (https://miriade.eu/) has gathered expertise to review the technologies that are currently available in research settings and, in some cases, in clinical laboratory practice, presenting a form of lexicon on each technology.

**Fig. 1:**
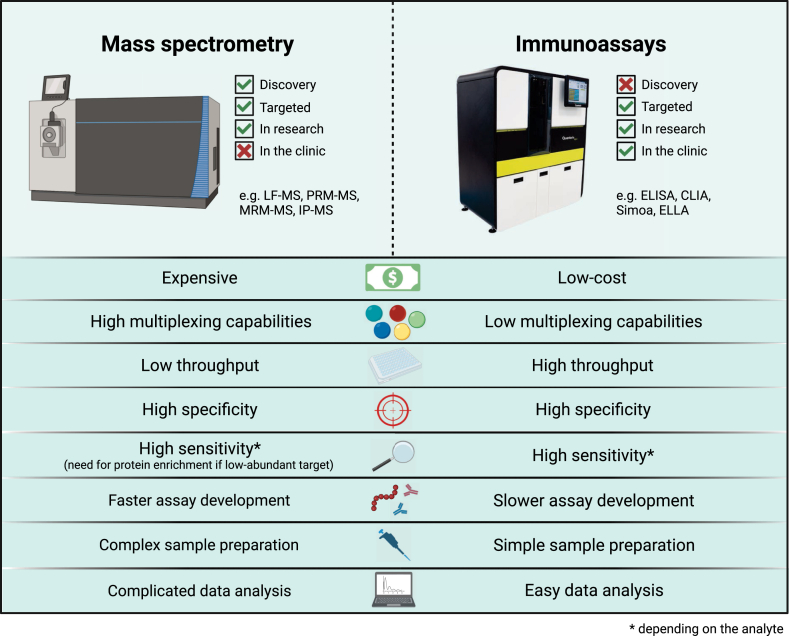
**Comparison of mass spectrometry assays *versus* immunoassay.** Conceptual characteristics that can be relevant for use in research or clinical practice are compared.

This lexicon lists currently available methods, broken down into mass spectrometry (MS)– and immunoassay-based methods, and reviews them in regard to analysis principle, required instrumentation, clinical and research use, strengths and limitations, and future perspectives. The aim of this review is to give the reader a complete overview of the toolbox for biomarker discovery and validation in the field of neurological disorders. The reader will become familiar with established and new technologies for both global/omics approaches and targeted analysis of biomarkers (Fig. 1). Furthermore, the lexicon may constitute a reference to understand the strengths and limitations of each methodology and how these can be used in a complementary fashion to answer specific research and clinical questions.

## Mass Spectrometry–Based Methods

Mass spectrometry approaches are increasingly used in the context of research or clinical practice for biomarker discovery and validation. While immunodetection studies use protein-specific antibodies to selectively isolate a protein of interest in complex mixtures like biological fluids, MS–based assays can analyze the protein content of the sample without the need for antibody-based enrichment (although combinations in the form of immunoprecipitation MS exist). Samples can be proteolytically digested (bottom-up approach) or used intact without previous proteolytic cleavage (top-down approach). Two complementary MS applications are used: large scale proteomics for biomarker discovery and targeted approaches for biomarker validation.

## Large-Scale Quantitative Mass Spectrometry

Label-free mass spectrometry (LF-MS) workflows use an untargeted approach to analyze the proteome content in a variety of biological fluids and samples (9). The LF-MS workflow uses a ‘bottom-up’ approach and is commonly used for large scale proteomics study. Proteins are extracted from samples and digested using enzymes called proteases. Resulting peptides are separated with liquid chromatography methods and analyzed by MS (9). LF-MS does not require chemical labels or an internal standard.

A variety of mass spectrometer platforms with different ionization modes, mass analyzers, and detector types can be used for LF-MS proteomic analysis (10). The most widely used ionization technique is electrospray ionization and mass analyzers that can achieve high accurate mass include orbitrap, quadrupoles, and time-of-flight. High-resolution accurate mass instruments represent the current state-of-the-art analyzers for quantitative proteomics (11). The bottom-up approach is referred to as peptide-centric, as identification and quantification is carried out at the peptide level (11). Protein inference is then performed to obtain a cumulative signal representative of the quantitative levels of the proteins analyzed. Data acquisition is currently based on two main paradigms: data-dependent acquisition (DDA) and data-independent acquisition (DIA). In DDA, the most abundant signals (Top N) are recorded in each MS scan, whereas in DIA, for each MS scan, a set of acquisition windows is used to record all theoretical signals and subject them to further fragmentation and detection (12). These methods require different search strategies for peptide/protein identification. For DDA, the mass signals of each peptide fragment are matched to theoretical masses coming from an “*in silico*” digestion of a protein database. In DIA MS, the complex data pattern is deconvoluted using experimental spectral libraries, that is, a collection of real spectra usually built from the same sample type analyzed or pan-libraries including spectra collected from different biological fluids or tissues (12). With libraries, the search space is limited to the peptide fragments included in the libraries, but the information about fragment intensity is retained whereas it is usually lost in DDA methods. New approaches can also use specialized computational methods to match and quantify DIA experiments without the use of spectral libraries (13). Depending on the acquisition methods, several quantification workflow methods have been developed over the years; some are based on spectral counts (*i.e.*, the number of spectra for each protein) (14), but the majority of methods currently in use are based on the intensity quantification of MS1 (precursor) or MS2 (fragment) spectra, the latter often being used in DIA approaches (12).

## Targeted Quantitative MS

Similarly to large scale proteomics studies, targeted quantitative MS approaches focus on free peptides or the proteolytic digest of the entire protein repertoire of a sample. As such, in generic MS approaches, the high dynamic range of protein abundances impedes the quantifiability of low-abundance proteins. Particularly in clinical neurology, ultrahigh sensitivity is indispensable since brain-derived proteins that are present at very low concentrations in blood may be valid biomarkers for a specific neuropathology (15).

The most straightforward approach of MS relies on multiple reaction monitoring (MRM) and parallel reaction monitoring (PRM) which are targeted ion-monitoring techniques. While PRM is typically performed on high-resolution accurate mass instruments such as quadrupole-Orbitrap hybrid or Q-ToF systems (15), MRM is often applied on low-resolution triple quadrupole (QqQ) mass spectrometers. Both in MRM and PRM approaches, proteins are quantified by the detection of proteotypic peptides, which are unique representatives of a single protein, rather than detecting the entire protein. Sample matrices are subjected to protein denaturation, reduction, and alkylation, followed by enzymatic digestion of the protein, usually using trypsin, into peptides. To limit ion suppression in the ionization source, peptides are separated by LC prior to MRM-MS/PRM-MS analysis. LC is coupled to an electrospray ionization source to ionize the peptides and to transition them into gas phase while entering the mass analyzer.

In MRM-MS, the first quadrupole can separate peptides based on mass-to-charge (m/z) and selects the m/z of the proteotypic peptide of interest (precursor ion). In the second quadrupole, or collision cell, the peptide is fragmented by collision-induced dissociation with an inert gas (nitrogen or argon). The third quadrupole functions as another m/z filter through which preselected fragment ions from the precursor ion pass and are detected. Thus, MRM-MS filters the m/z at two levels, significantly reducing noise while increasing sensitivity. Therefore, selection of the precursor ion and its associated fragments is a crucial step in the development of an MRM-MS assay (16).

Similarly, in PRM-MS, once the sample has been ionized and injected into the mass spectrometer, which is often an Orbitrap instrument, one or several precursor ions of interest are selected according to the set m/z ratio in the quadrupole chamber, followed by its fragmentation by higher energy collision-induced dissociation in the collision cell and finally analysis in the Orbitrap. Over the course of the elution time window (predetermined in the optimization step of the method), apart from the precursor ion, the mass analyzer acquires all the MS/MS spectra corresponding to each of the fragment ions (15).

Quantification in both of these targeted approaches can be performed by spiking samples with an isotopically labeled protein or peptide and monitoring both the endogenous peptide and the isotopically labeled peptide simultaneously (17).

Targeted assays are therefore designed with a specific hypothesis in mind, meaning that the protein of interest and its proteotypic peptides must be determined in advance, to create a specifically targeted method (18). The natural workflow of such an assay is:

1. Biological question: Is the protein of interest putatively altered in a certain condition?
2. In silico selection of proteotypic peptides (parent ions) from the protein of interest and their transition (fragment ions) using specific software as Skyline MacCoss Lab Software. Selection of fragment ions is only necessary for MRM-MS as PRM analyze all fragments ions.
3. Determining the m/z ratio of precursor ions and fragment ions, collision energy, and retention time of the peptides.
4. Analyzing samples by MRM-MS/PRM-MS.

Interestingly, sample preparation is a major component of MS, particularly when applied to biofluids. The separation of peptides using LC and reversed-phase columns (C18) is, on some occasions, insufficient to prevent ion suppression of low-abundance proteins by high-abundance proteins. Sample preparation techniques can be applied prior to protein digestion (*e.g.*, protein depletion, protein precipitation, or immunoprecipitation) and/or post-digestion (*e.g.*, peptide fractionation) to simplify the biological matrix or to enrich the protein or peptide of interest and enhance its detectability.

In some instance, the sensitivity of the MRM/PRM approach even with complex fractionation procedures before MS is not high enough to capture the most specific and low concentration targets, especially in the blood. In this case, immunoprecipitation mass spectrometry (IP-MS) that combines antibody-based enrichment of the target protein from the sample with the ability of MS to accurately quantify the protein or provide structural information can be used. For immunoprecipitation purposes (*e.g.*, protein purification), antibodies are usually bound to magnetic beads that are added to the sample, followed by an incubation step in which the protein of interest binds to the bead-bound antibodies. The beads are then washed, followed by protein recovery using a low pH solution to disrupt the interaction between the antibody and the bead. The enriched proteins are then subjected to tryptic digestion, followed by quantification with LC-MS/MS.

## Applications in Research

The combined use of targeted and untargeted MS approaches is a powerful tool for biomarker discovery and validation. Untargeted approaches alongside bioinformatics workflows allow unbiased identification of protein profiles specific to disease state. Candidate biomarkers can be identified and submitted to specific targeted MS workflows. Targeted workflows allow greater specificity and sensitivity.

LF-MS workflows can be used for biomarker discovery where samples are screened to select suitable candidate peptides. A validation sample set can be assessed containing larger number of samples and additional orthogonal techniques (19, 20, 21). Validation can be performed using MRM-MS or PRM-MS, where low-abundant proteins can be detected and quantified in a reproducible manner with greater sensitivity (22, 23).

LF-MS studies have characterized the proteomes of biological fluids and brain tissues taken from patients with diagnosis of different neurodegenerative disorders including AD, Parkinson’s disease (PD), FTD, dementia with Lewy bodies (DLB), and ALS (19, 20, 21, 24, 25, 26). These studies examined the protein expression profiles from cohorts of patients with well-characterized disease states with matched controls with the aim to find specific biomarkers for each neurodegenerative disorder. Changes in proteins expression could be mapped and linked to pathophysiological mechanisms (27, 28).

An important development has been the characterization of protein deposits in neurodegenerative disorders. A recent study evidenced how amyloid plaques in addition to amyloid peptides have enrichment in several other proteinaceous components, some of which are shared between AD and Down syndrome (29). LF-MS has been utilized to study Lewy bodies (30, 31), and the extracellular vesicles proteome (32). By targeting subproteomes (26), specific proteins may be enriched and associated with certain subcellular structures or organelles in disease states. These powerful techniques can be used to facilitate biomarker discovery and the investigation of specific pathophysiological mechanisms in the translational studies of disease models and patients.

In contrast to LF-MS methods that quantify relative changes in patient samples, both PRM and MRM determine protein levels with absolute quantification. For example, an MRM-MS assay was developed to evaluate α-, β-, and γ-synuclein levels in the CSF of patients with different synucleinopathies (33). Another powerful feature of MRM-MS is the capability to differentiate between isoforms. For example, apolipoprotein E (ApoE) isoforms, which only differ by one or two amino acid substitutions, were individually quantified with this approach in blood and CSF (34, 35). The isoform ApoE4 is a known genetic risk factor for AD. Similarly, tau protein, which is associated with tauopathies such as AD, is characterized by a high molecular diversity at the posttranslational and isoform-level. Tau protein was quantified in CSF using an MRM method to monitor seven peptides of the protein previously validated by PRM. However, this assay measured the total tau concentration and did not differentiate the phosphorylated isoforms of the protein, which are known to be more predictive (36). Enrichment of the phosphoforms would be necessary. Similarly, another study performed PRM to monitor 13 different proteins based on their association with neurodegenerative diseases, assessing their value as biomarker candidates (23).

IP-MS represents a good alternative to detect low abundant targets of clinical interests. As an example, an IP-MS approach to quantify in the blood Aβ peptides (37) (and combined with ApoE (38), this approach has been approved for clinical use). An IP-MS approach allowed the quantification of the presynaptic protein SNAP-25 in CSF, which is a specific marker for AD (39).The clinical performance (ROC curve and fold changes) of the IP-MS assay to differentiate between AD and controls with SNAP-25 was recently shown to be similar to that of a single molecule analysis (Simoa) assay (see chapter below) for the same biomarker (40). The development of an IP-MS assay for beta-synuclein significantly advanced the field of synaptic markers in AD by identifying elevated levels in both CSF and blood of patients with AD (41). IP-MS was also applied in a study targeting tau protein in blood and CSF, allowing identification of truncated forms and quantification of, for example, pTau181 and pTau217, which are highly specific biomarkers for AD (42, 43). A variant of immunoprecipitation is stable isotope standards and capture by anti-peptide antibodies (SISCAPA) (42). SISCAPA-MS differs from the conventional IP-MS workflow by first digesting the proteins, followed by capturing proteotypic peptides using antipeptide antibodies. SISCAPA-MS has been used to quantify leucine-rich repeat kinase 2 (LRRK2) in CSF, showing increased levels in PD patients with the G2019S mutation (44).

## Applications in Clinical Practice

On its own, MS is the technique of choice for routine targeted analysis of small molecules like metabolites and drugs. Both MRM and PRM approaches can be applied to selectively quantify biomarkers in neurodegenerative diseases but can also be valuable for the standardization of available clinical assays. For instance, MRM-MS was used for the standardization of measurement and the development of certified reference materials for Aβ42 in CSF (45, 46). Additionally, untargeted LF-MS is still difficult to apply in clinical settings due to some of the limitations described below and is mostly suited to discovery settings.

## Strengths

The ability of MS to differentiate between protein isoforms and posttranslationally modified proteins, in addition to its high multiplexing capabilities, can significantly increase the amount of biological information obtained from patient samples. MS methods do not rely on antibodies, which can be an advantage of commercial antibodies against the target protein which do not exist or are hard to generate. Additionally, the high specificity is an attractive feature of MS for biomarker screening, clinical validation, and assay standardization (47).

The major advantage of LF-MS is the simplicity of the experimental setup with no need of labeling and subsequent complex experimental design. Depending on the type of sample, the depth of analysis can be pushed up to 10,000 proteins per run (48, 49). This number is usually lower in biological fluids where high abundance proteins can mask protein biomarkers present at lower levels (50). Using sample fractionation, it is possible to identify about 3000 proteins in biological fluids like CSF (51), whereas this number is about 500 to 1000 proteins per run without fractionation (50). LF-MS is a powerful approach for discovery proteomics as quantification of protein levels can be accomplished at a global level. Importantly, the technique is applicable to a wide variety of samples (cell culture, biological fluids, tissue extract, etc.) by adapting the protein extraction protocol.

Recent advances using DIA methods have overcome some limitations such as the low reproducibility of proteins quantification in DDA approaches. In particular, the use of DIA methods and optimized LC enable thousands of proteins to be quantified in only 5 min (52) with coefficients of variation comparable to those of immunoassay methods.

An advantage that PRM has over MRM is that all fragment ions of each peptide are analyzed and stored, meaning the selection of a relevant transition for quantification can be made retrospectively, with no need for selection prior to analysis. In addition, PRM assays are typically performed in quadrupole-Orbitrap hybrid instruments in which the mass analyzer, the Orbitrap, offers greater resolution and therefore more specificity than MRM assays, which are performed in triple quadrupole instruments (53). The strength of IP-MS is based on the enrichment of the protein of interest using immunoprecipitation prior to MS, which significantly simplifies the matrix allowing detection of proteins, even when present in minute amounts. Interestingly, even if the antibody used to capture the target is not specific, the following MS detection is by nature highly specific and can detect specific proteoforms. Furthermore, enrichment methods allow the analysis of a wide range of PTMs including phosphorylation, ubiquitination, glycosylation, acetylation, and others (54).

## Limitations

A drawback of using quantitative MS for protein quantification is the need to enzymatically digest the protein, usually using trypsin, which cleaves peptides flanked by either lysine or arginine, thus limiting the available peptides in a protein that can be measured. In addition, some of these peptides may not be suitable for LC-MS.

One of the main limitations of LF-MS is its poor reproducibility, especially when DDA methods are used. Coefficients of variation between 15 and 20% were obtained for technical replicates, with a large percentage of missing values (4–20% of the total identified proteins, depending on the type of samples) (55). This is due to the stochastic approach of DDA acquisition in which only the most intense precursor ions are selected (12). The relatively low reproducibility also results from the coupling between LC and MS. It was demonstrated that although the LC step is necessary to reduce sample complexity, it may introduce variability in the identification and quantification of peptides and proteins (56). Sample separation using LC methods reduces the throughput of the technique as samples must be run sequentially. The average time for each sample is 1 to 2 h depending on the type of chromatography and acquisition method (56).

Differential expression analysis can also be less precise than label-based approaches, in which the presence of a labeled counterpart of each peptide can further contribute to the accuracy of the quantification (57).

Other limitations of MS are related to the complexity of the MS technique. To obtain reliable results, highly trained personnel is needed, together with dedicated equipment and instrumentation that can be available only in specialized laboratories.

## Future Perspectives

In the future, LF-MS will continue to play a role in biomarker discovery and clinical applications for neurological diseases thanks to the technological improvement and the availability of faster and more sensitive MS instruments (50). Nowadays, it is possible to analyze large cohorts of patients (>1000) minimizing the sample turnaround and obtaining high quality data also on challenging biological fluids like plasma (51). Additionally, the possibility to identify and quantify posttranslationally modified proteins and peptides (58) may contribute to the discovery of specific PTM signatures for neurodegenerative disorders as modifications of proteins like amyloid or synuclein are closely linked to the pathogenetic processes.

Several publications have shown the superiority of quantitative targeted MS methods over immunodetection for key blood biomarkers of AD, such as amyloid and tau proteins (59). Implementing these approaches for clinical application is therefore an attractive perspective that will, however, require significant evolution in technology and cost to be compatible with routine use. The prospect of using the multiplexing capacity of MS to quantify a panel of biomarkers is also very interesting. One can imagine combining biomarkers addressing different aspects such as neurodegeneration, neuroinflammation, synaptic function, and co-pathologies. It will then be possible to establish efficient and reliable algorithms combining these markers.

## Antibody-Based Detection Methods

Antibody-based detection methods are widely applied in routine clinic practice. They have proven value for large scale analysis and in principle offer a strong sensitivity and specificity for the targeted proteins in their native configuration. Originally used for single protein analysis, nowadays, more multiplexing methods are available, covering a large portion of the human proteome.

## Proximity Extension Assay–Based Proteomics

The proximity extension array (PEA) technology is a multiplexed antibody-based proteomics method, first described in 2014 (60), nowadays allowing to detect over 3000 proteins. It combines antibody- and DNA-based methods to measure protein levels in different body fluids, such as blood and CSF. Each target protein is detected by two antibodies that are coupled to unique DNA oligonucleotide sequences, specific for each target protein. Upon binding of both antibodies to the target, these DNA oligonucleotide sequences are in close enough proximity to hybridize. In the next step, a DNA polymerase extends the hybridized DNA template into a unique dsDNA sequence. The amount of dsDNA is then proportional to the concentration of the target protein in the sample. Lastly, the unique dsDNA sequences are amplified by PCR and quantified by either quantitative real-time PCR or next generation sequencing (60).

## Applications in Research

In neurodegenerative dementias, the PEA technology has been used to identify novel plasma and CSF biomarkers, for example, for the differential diagnosis and prediction of conversion to dementia (61, 62, 63, 64, 65, 66, 67). To characterize the disease biology, multiple studies focussed on analyzing various disease mechanisms, such as inflammation (62, 68, 69). Altogether, these applications can also aid the search for novel drug targets for neurodegenerative diseases (2, 62, 68, 69, 70).

## Applications in Clinical Practice

To date, there is no application for the PEA technique in the clinic. The platform is used for discovery purposes and disease modeling. This may be due to the fact that only relative protein concentrations are being measured, which do not allow for the establishment of protein-specific cut-off values (some PEA methods are now being calibrated against protein standards, wherefore this may change soon).

## Strengths

Strengths are that through multiplexing, several thousand proteins can be measured simultaneously in one sample, which requires a total volume of only 3 μl (71). Due to the requirement of two matching antibody-DNA pairs and protein-specific primers used for dsDNA amplification, the frequently observed cross-reactivity effect during multiplexing is minimized.

The amplification of dsDNA sequences using PCR allows for the detection of proteins at very low concentrations, thus increasing sensitivity (72). By using antibody-based methods for biomarker discovery, the technology translation gap to single immunoassays is overcome. With Olink proteomics for biomarker discovery, the same antibody pairs applied in the discovery panels can be used for further single biomarker assay development.

## Limitations

A limitation for biological enrichment analyses is that the protein selection is biased to proteins with good antibody pairs available, possibly overestimating those proteins with good antibodies available and underrepresenting those without or with only weak antibodies available. Furthermore, only a relative quantification of protein concentrations is provided for most panels, not allowing for the direct comparison of different protein levels in one run or the same proteins between runs without the use of bridging samples (72).

## Future Perspectives

Identification of novel proteins and development of highly specific antibodies will allow for the further extension of the PEA panels. Additionally, smaller custom panels, including protein standards for absolute quantification, can be generated to target specific diseases. With increasing numbers of studies using PEA becoming available, cross-disease meta-analyses will become important. To create more depth of the interrogation of the proteome, studies have started to use multiplatform proteomics, combining PEA, SomaScan, and MS proteomics (73).

## Multiplex Protein Analysis Using Bead-Based Assays

Another multiplex analysis strategy based on antibody binding is bead-based microarrays such as provided by the Luminex Inc platform, enabling high-throughput multiplex protein analysis. Here, captured antibodies are immobilized onto the surface of magnetic and color-coded beads, which are then mixed to create a suspension bead array. The color codes provide each bead with an identity, enabling identification of the specific antibody-target interaction upon readout. This allows for simultaneous analysis of multiple proteins in a single well of a microtiter plate. In these assays, the detection of proteins is mediated through direct labeling of the protein content in each sample. One such labeling strategy is to use (+)-biotin N-hydroxysuccinimide ester that covalently links to the primary amines of the proteins. The biotin molecules can thereafter mediate protein detection using a streptavidin-conjugated fluorophore. Even though multiplexing is limited by the number of unique bead identities available, established protocols are available for both CSF and plasma analysis with the capacity to multiplex up to 384 targets in parallel analysis of 384 samples (74, 75).

## Applications in Research

Bead-based protein profiling assays are widely used in various research settings. For example, CSF levels of the core AD biomarkers have been analyzed in a multiplex fashion both using in-house developed methods (76, 77), as well as commercially available kits (78). There are also examples of how predefined cytokine panels have been used for serum analysis in the context of PD (79) and complement factors in plasma in search for FTD-associated profiles in samples from the GENFI consortium (80). In one study, a custom-designed panel was assembled based on initial analysis of a predefined set of proteins and evaluated in serum samples from an AD cohort (81). The single binder assay has been applied to perform large-scale analysis of both CSF and plasma/serum in the context of AD (82, 83), FTD (84, 85), ALS (86), and for the analysis of CSF protein levels in cognitively healthy individuals (87). These studies have reported strong associations with disease diagnosis, as well as with subgroups and clinical characteristics of patients.

## Applications in Clinical Practice

Some bead-based assays are used in clinical laboratory practice, for example, in clinical immunology, but, in general, multiplexed analysis methods using bead-based assays have mainly been limited to research use, although various translational approaches are being developed.

## Strengths

Classical antibody-based methods are suitable for targeted investigation of specific proteins but are not optimal for wider explorative approaches. The main strength of bead-based multiplex immunoassays is the capability of a targeted setup enabling both high-throughput and multiplexed protein profiling. Strengths also include the low sample consumption; for the single binder setup, as little as 15 μl of CSF and 3 μl of plasma is required for labeling, and the labeled volume can be utilized for analysis of up to six different 384 protein assays. Sample preparation and workflow procedures are commonly kept simple and possible to perform with standard lab equipment but can also be implemented with robotic handling and automation. Due to the use of antibodies, the methods can be applied also on samples of high complexity while still retaining relatively high sensitivity and specificity. The limit of detection depends on the specific reagents used but generally range between low pg to low ng/ml (88). Standard curves can be included for singleplex assays, allowing for absolute quantification estimations and combination of datasets generated over long periods of time. Protein panels can be specifically defined for each research question and study. It is also possible to convert from the multiplexed and exploratory study setup to more focused validation studies using the same set-up of reagents.

## Limitations

The multiplexing nature comes with a trade-off in terms of optimal analytical conditions for the included proteins. Samples are treated equally for all proteins in a panel and the conditions may not be optimal for each individual reagent. Furthermore, standard curves cannot be included for all proteins in large-scale studies. In commercial assays, the cost is based on the full panel, so if only a few proteins are of interest in the context of a specific study, the analysis can be perceived as expensive.

The inclusion of proteins in a panel is based on availability of suitable antibodies. In-house creation of larger panels could therefore be restricted to research environments with access to large numbers of antibodies such as within the Human Protein Atlas (www.proteinatlas.org). Assay performance depends on antibody specificity and selectivity, and in single binder setups, where only one antibody is used for protein detection, any unspecific interaction will generate a signal (which would have been canceled out in a sandwich format where two antibodies specific to different epitopes on the same protein are used). As with all immunoassays, results need to be validated in terms of both biological and technical reproducibility.

## Future Perspectives

The availability of antibodies is expected to increase with time, further expanding the existing panels and broadening the application for multiplexed assays. Both sensitivity and multiplexing capacity can be improved through development of new detection methods (*e.g.*, light-initiated chemiluminescence) (89) and clinical applications using protein panels will likely emerge in the coming years.

## Enzyme-Linked Immunosorbent Assay

An ELISA is an antibody-based technique to detect trace proteins in a liquid matrix. First developed in 1971, ELISA is a member of the second generation of quantitative diagnostic tools used in medicine (58, 90). The key step of an ELISA involves the immobilization of the antigen of interest on the surface of a microplate that can be detected by a direct or indirect enzymatic reaction. The immobilized antigen can be detected using a primary antibody conjugated to an enzyme (direct ELISA) or an unlabeled primary antibody conjugated to an enzyme-labeled secondary antibody (indirect ELISA). Another widely popular and highly sensitive format is the sandwich ELISA where the captured antibody is immobilized on the surface of the microplate. The antigen binds to this capture antibody and an enzyme-coupled secondary antibody. The enzyme-substrate reaction generates a chromogenic or fluorescent read-out that is directly proportional to the amount of antigen present (91).

## Applications

Immunoassays, particularly ELISA, play a key role in clinical medicine for the diagnosis and prognosis of diseases. In the field of neurodegenerative disorders such as AD, low-abundance proteins present in the CSF of patients are often quantified using ELISA (92). Some examples of CSF biomarkers routinely quantified using the ELISA technique include Aβ (Aβ40 and Aβ42), total tau and phosphorylated tau, neurofilament light chain (NfL), and neurogranin (93, 94, 95, 96). Often, the clinical relevance of novel biomarkers discovered through proteomics techniques is evaluated using ELISA as a first step. This is because ELISA is a robust, easy-to-use, and highly cost-effective analytical tool (90, 91).

## Strengths and Limitations

While the ELISA offers sufficient analytical sensitivity to measure trace neuronal proteins in CSF, it often lacks sensitivity to measure these proteins in the blood (58). It is also prone to handling errors, and optimization of pairs of antibodies suited for an ELISA often takes a long time (92).

## Future Perspectives

Despite the popularity and ease of use of ELISAs, the development of novel ultrasensitive immunoassay techniques has led to tremendous improvements in the analytical sensitivity (see below, Simoa and ELLA). Such novel and innovative immunoassay technologies offer higher sensitivity, accuracy, and more efficient sample measurement. While the predominance of ELISA in clinical diagnostics is unlikely to see a sharp decline, the future of *in vitro* diagnostics industry lies in digitalization and multiplexing of immunoassays.

## Chemiluminescence and Electrochemiluminescence

The chemiluminescent immunoassay (CLIA) or electrochemiluminescent immunoassay are closed system antibody-based assays where the indicator of the analytical reaction is luminescence (97). In both techniques, the immunocomplexes are captured *via* a biotin-streptavidin binding on magnetic beads, but they differ in the detection system, that is, for ECL, this is based on ruthenium-coupled antibodies, while CLIA uses alkaline phosphatase–coupled antibodies or their derivatives (98). Direct CLIA methods make use of lumiphore markers while indirect techniques use enzyme markers (97).

## Applications

The CLIA technology is used in the Lumipulse (Fujirebio) and the HISCL (Sysmex) instruments (99, 100). The ECL technology, on the other hand, finds its use in the MesoScale Discovery (Lilly Research Laboratories) and Elecsys (Roche) analytical systems (98, 101). In clinical neurochemistry, these two techniques are applied for the diagnosis for AD. The measurement of Aβ and phosphorylated tau in CSF of patients are routinely carried out using the Elecsys and Lumipulse platforms, while the Mesoscale Discovery has been instrumental in the establishment of phosphorylated tau measurements in plasma (45, 102, 103). Similar assays are in development on the Elecsys and Lumipulse platforms as well.

## Strengths and Limitations

A major advantage of luminescent methods over colorimetric methods such as ELISA is that the luminescence is an absolute measure of the analytical reaction, while the latter measures the product of an enzyme reaction, which is a relative measurement (this is also relevant for some of the CLIA methods). Other advantages of this technique include its large dynamic range as well as high sensitivity and specificity. There are, however, several limitations to the CLIA technology. These include its higher cost compared with an ELISA, limited availability of detection of analyte and testing panels, and closed analytical systems (97).

## Future Perspectives

The CLIA shows great potential to be used for multiplexed immunoassays, which are gaining popularity for clinical applications. For example, multianalyte CLIAs are available using an array-based technique where different antigens are immobilized onto a solid phase such that multiple reactions may occur simultaneously, for example, as in the Mesoscale Discovery System. It is expected that with the evolution of these technologies, the translation of novel biomarkers into clinical use will be expedited.

## Novel Methods for Effective Antibody Generation

The implementation of fluid biomarkers for clinical research is often a slow and tedious process, one of the major hurdles being the translation of novel biomarker candidates into sensitive immunoassays. Thus, the efficient generation of highly specific novel antibodies is an essential first step towards the development of high-throughput antibody-based assays (104). The advent of advanced bioinformatics and artificial intelligence tools has simplified this process as it is now possible to sequence millions of proteins simultaneously, as well as predict their structures (105, 106). To generate antibodies with high affinity for the corresponding antigen, these prediction tools may be applied for understanding the antibody conformation and geometry. When these antibodies are raised in animal models using hybridoma technology, computationally modeled immunogenic peptide immunizations are often used. Examples of such conformational peptide design for antibody generation include oligomer-specific antibodies against Aβ and alpha synuclein (107). While the market of antibodies is still heavily reliant on the use of such animal models, scientific advancements now allow us to generate *in vitro* recombinant antibodies with well-defined sequences (108). Research has also shown that the use of monoclonal antibodies for the development of immunoassays improves their robustness and reproducibility (109).

## Simoa

Simoa is a bead-based ELISA run on the automated HD-X/HD-1 or manual SR-X instruments, provided by Quanterix. Simoa sandwich immunocomplexes are formed by incubation of the following: (1) a bead-conjugated capture antibody, (2) samples containing the target protein, and (3) an enzyme-labeled detector antibody. The mixture of bead-conjugated immunocomplexes and an enzyme substrate is transferred to microarray discs with 50-fL wells that are sized to confine only one bead. Once the bead-coupled immunocomplexes are loaded to wells and sealed, fluorescence of single beads is read, as the enzyme converts the substrate into a fluorescent reaction product. The Simoa software calculates the fluorescent measurements as average number of enzymes per bead (AEB), which can be further translated to concentration units using a calibration curve. At low concentrations of target protein, the reaction environment includes much more beads than protein molecules; hence, following the Poisson distribution, beads carry no or only one enzyme. Therefore, at low concentrations, AEB is determined by the digital count of positive beads, defined as those emitting fluorescence. At higher concentrations of a target protein, more enzymes can be bound to single bead; the AEB is computed based on the averaged analog signal output from all the beads present on a microarray.

## Applications in Research

In recent years, multiple research groups implemented Simoa to evaluate diagnostic potential of classical dementia biomarkers measured in matrices alternative to CSF, such as blood plasma (110, 111) or saliva (112). In the field of AD research, the most relevant steps toward CSF-to-plasma shift include Simoa measurement of both classical (Aβ42, Aβ40, p-tau181, p-tau217, p-tau231, and total tau) as well as emerging, complementary (NfL, GFAP) biomarkers in plasma (113). In addition to the use of Quanterix-offered kits, diverse homebrew Simoa assays were established and validated in relevant patient cohorts and, in selected cases, also commercialized (114, 115, 116, 117).

## Applications in Clinical Practice

Although the current Simoa portfolio consists mainly of Research Use Only assays, the technology may enter the field of dementia diagnostics as the first Laboratory Developed Test for the measurement of plasma p-tau181 has been recently launched (114, 115, 116, 117), (https://www.quanterix.com/press-releases/quanterix-launches-first-ptau-181-plasma-laboratory-developed-test-for-clinical-diagnostic-and-research-applications-in-the-us/). Additional Simoa assays, for example, plasma NfL, have also been validated for clinical use in countries where this is allowed.

## Strengths

An indisputable strength of Simoa is its sensitivity, often being 1000x higher than a conventional ELISA, which is crucial for the field of neurodegenerative dementias where many target proteins are present at extremely low concentrations (118, 119). A clear illustrative example of this is that in a platform comparison study for the neurodegenerative biomarker NfL, where all three platforms employed the same antibody pair, only with the Simoa NfL assay a significant difference in plasma levels was observed between healthy controls and patients with multiple sclerosis (119). Thanks to the combination of digital and analogous read-outs, Simoa provides also a wide dynamic range. Among other strengths of Simoa is that the technology provides high throughput (up to 288 data points per single run when run on the HD-X/HD-1), which is essential when considering the platform for large batch analyses. In addition to a wide portfolio of robust assays, Simoa enables the design of homebrew assays. In the workflow of dementia biomarker development, in-house–developed Simoa assays may play an important role as a tool for validation of new candidate biomarkers discovered through proteomics studies (120). Lastly, in addition to singleplex assays, multiplex assays can be run, enabling simultaneous measurement of several analytes of interest in a single test and in low sample volume (121, 122). Multiplexing is beneficial considering the multifactorial nature of many neurodegenerative diseases, such as of AD, where it was shown that measurement of a combination of markers improves diagnostic performance (110).

## Limitations

The factor most significantly limiting overall availability of Simoa is the high cost of instruments as well as dedicated consumables and reagents. Additionally, specialized staff is needed to ensure proper operation and maintenance of platforms.

## Future Perspectives

Recent study reports further optimization of Simoa obtained by improvement of bead read efficiency. The modified technology allows for detection of proteins at as low as sub-attomolar concentrations; such sensitivity, if available on the market, could open a window of opportunity for biomarker measurement in new matrices (123).

## Microfluidic Immunoassay (ELLA)

The ELLA platform is a microfluidic immunoassay system provided by ProteinSimple/Bio-Techne (124). The ELLA cartridges use microfluidic channels in which three glass nanoreactors (GNRs) are located. The GNRs are coated with a capture antibody which binds the target analyte. After the removal of unbound analytes, a detector antibody is flown through, and using a fluorescent detection system, triplicate results are produced for each sample due to the three GNRs per channel. Concentrations are generated by using the factory-calibrated standard curve already on the cartridge. The system can be used for single and multiplex measurements. Furthermore, there is the option to establish in-house ELLA assays using uncoated open cartridges.

## Applications in Research

ELLA cartridges are deployed for a wide range of biomedical research fields, such as neuroscience, cancer, COVID-19, and inflammation. In neuroscience biomarker research, ELLA assays are validated in CSF for 26 proteins, including NfL, neurofilament heavy (NfH), chitinase 3-like 1 (CHI3L1), and GFAP. Moreover, most proteins like, for example, NfL and NfH can also be detected in blood.

## Applications in Clinical Practice

As the ELLA platform is relatively new, there are so far no applications in neurological clinical practice. However, the analysis of NfL and NfH in CSF and blood runs robustly (125). In the future, these assays could be applied, for example, in the diagnostic workup of ALS.

## Strengths

The ELLA platform is easy to use, generates fast results, and yields robust data. In addition, it is a small benchtop machine that can be easily applied in daily routine in the clinic. The validation of proteomic data, for example, novel candidate biomarkers, using different technology, is a highly important step toward new biomarkers for neurological diseases. For this purpose, the ELLA open-cartridge version allows users to set up their assays. This is crucial as identified candidate biomarkers are often not commercially available as ready, easy-to-use, high-throughput immunoassays.

## Limitations

The downside of the ELLA platform research-wise is the mandatory use of the complete cartridge without being able to save wells for consecutive runs. Moreover, the costs per sample are more expensive than general ELISAs. Regarding assay performance, ELLA assays are highly sensitive with lower limits of detection in the low or even sub-picogram range; however, in the case of NfL and some cytokines, do not quite reach the sensitivity of the Simoa Bead technology (126, 127, 128).

## Future Perspectives

In the future, the small and easy to use benchtop ELLA platform could be applied in daily clinical routine to analyze low-abundant biomarkers helping clinicians in the diagnostic workup of neurological diseases. In addition, it might also be feasible to apply the ELLA technique for point-of-care testing directly in the emergency room or for patient monitoring at the bedside of intensive care units.

## Aggregation Methods (Seed Amplification Assay)

Other relevant protein detection methods for neurodegenerative diseases are assays to sensitively detect protein seeds or aggregates. Initially, the assays were introduced as protein misfolding cyclic amplification in 2001 (129) or real-time quaking-induced conversion (RT-QuIC) in 2010 (130, 131). Considering the similarities between the techniques, these assays are now collectively referred to as seed amplification assays (SAA) (132). SAA is currently used to detect very low concentrations of amyloid fibrils in human biospecimens. In SAA, a biological matrix (fluid or tissue) is incubated in the presence of a reaction mix containing the monomeric form of the specific amyloidogenic protein. If present, fibrils then catalyze the misfolding of the monomeric substrate which results in elongation of the fibrils. The application of cycles of vigorous shaking/sonication promotes fragmentation of the fibrils thus enhancing the amount of fibrillar ends that can be elongated by new monomers. These steps lead to an exponential growth of the fibril ends and hence of the total protein mass in amyloidogenic form. In most SAA protocols, the whole aggregation process can be monitored in real-time by recording the fluorescence of thioflavin-T, a fluorophore having a high affinity toward amyloidogenic aggregates (129).

## Applications in Research

Although SAA was first successfully used for detecting human prions, they are currently also being applied for the detection of prion-like aggregates (132, 133, 134). Among all the relevant clinical applications, SAA demonstrated to be effective in detecting synucleinopathies. Indeed, α-synuclein SAA are capable of reliably detecting PD and DLB (135, 136); for a comprehensive review, see (134). The results show sensitivity for α-synuclein pathology in the preclinical phases (137). However, fewer protocols were also able to detect specific α-synuclein aggregates belonging to multiple system atrophy (138, 139, 140, 141). α-Synuclein SAA was adapted for various biological samples, including the brain, CSF, olfactory mucosa, skin, submandibular gland tissues, and saliva (134). More recently, there have been attempts also in developing tau SAA in postmortem brain and CSF samples. These assays succeeded in differentiating tauopathies with high sensitivity and specificity. Indeed, amplified tau filaments showed distinctive seeding activity in AD and chronic traumatic encephalopathy compared to other types of tauopathies. This led to the development of a 3-repeat/4-repeat tau test from brain tissue referred to as AD RT–QuIC 3R/4R (142). Recently, CSF tau SAA was developed for the 4-repeat (4R) tau aggregates of 4R tauopathies, namely progressive supranuclear palsy and corticobasal degeneration (143). In the context of ubiquitin-positive, tau-, and α-synuclein-negative FTD and ALS, so far, just one group attempted in developing a SAA protocol for the detection of misfolded TDP-43 (133).

## Applications in Clinical Practice

After years of harmonization and ring trial assessments (144), RT-QuIC has now become a reliable test, used to analyze CSF and olfactory mucosa, to support the clinical diagnosis of sporadic Creutzfeldt-Jakob disease (sCJD). CSF RT-QuIC positivity for prions has been included in the diagnostic criteria for sCJD (145), while protein misfolding cyclic amplification remained confined to the sole analysis of variant CJD (146).

## Strengths

The main strength of SAA is the ability to amplify even trace amounts of fibrils (less than a femtogram), which are not currently detectable by immunoassays. With respect to immunoassays, SAA do not suffer from cross-reactivity with monomers of the amyloidogenic protein of interest. SAA has the potential to not only increase our understanding of misfolded proteins but also help us diagnose α-synuclein pathologies at early stages (147).

## Limitations

Despite the many advantages of SAA, these techniques have shortcomings. The number of abnormal proteins that can be detected by RT-QuIC is currently limited. There is a lack of standardized protocols and reagents for α-synuclein and tau applications (134, 148) and many of the published protocols are time-consuming (from 2 to 5 days). Despite their high sensitivity and specificity in various biological specimens, SAA seems not to be applicable in blood, which is the most widely collected biological sample. Lastly, SAA techniques are so far not quantitative and cannot analyze gradual differences, which limits its application for treatment or disease progression monitoring.

## Future Perspectives

Currently, α-syn SAA in CSF and skin represents a promising tool for the identification of synucleinopathies, even at an early stage. To promote the use of α-syn SAA in the diagnostic workup of PD and related disorders, it would be desirable to improve protocol standardization and assay automation, as has been done in the context of AD biomarkers. The lack of quantitative response may currently prevent the use of these tests as an outcome measure in clinical trials with anti-α-syn drugs. Approaches such as kinetic trace fluorescence analysis of SAA and/or sample serial dilution allow to obtain a semiquantitative response, but further research is needed. In addition, the relationship and interaction with other biomarkers, such as other biomarkers of proteinopathies or biomarkers of neurodegeneration and synaptic dysfunction, and genetic status may require further investigation.

## Proteomics *Versus* Transcriptomics

Thus far, we have considered experimental techniques that probe the proteome. Here, we will also briefly consider if there is any value in considering the transcriptome in biomarker research for neurodegenerative disease. Proteomics is likely to be a more direct way to measure the changes in dementias than transcriptomics for the following reasons: (1) many dementias are thought to be largely driven by changes in the proteome and its interactions: proteins tend to aggregate, misfold, perturb membranes, change in terms of posttranslational modifications, and/or lose functionality within the disease pathology. None of these effects may directly be observed in the transcriptome. (2) In practice, it is not possible to obtain brain-derived cells from liquid biopsies (*e.g.*, from CSF or plasma) in conctrast to cancer, and hence it is difficult to probe mRNA expression in the brain *via* biofluids. Nevertheless, some immune cells, such as CD8 T-cells, can be derived from CSF (149).

## Transcriptome-Based Applications in Research

Despite proteome measurements being more direct, there are some important applications for the analysis of the transcriptome in the dementia field, in terms of biomarker discovery research as well as treatment. For proteins to be differentially abundant between healthy and diseased individuals, there can be multiple causes: (1) the abundance may be directly affected by the disease, for example, because the protein is no longer degraded, phosphorylated, or accumulating in an aggregate; (2) the disease may cause changes in gene regulation leading to changes in protein levels. Only the latter event may be probed by RNA-seq. It is therefore very informative to study both the proteome and transcriptome in parallel, as the multi-omics approach may reveal if the disease affects the brain on a gene regulation or protein level. For example, it has been shown that TREM2 is increased in sCJD patient brains at the mRNA and protein levels, while ADAM10 is increased at the protein, but not the mRNA level (150). Similarly, such discrepancies may also be probed in a systematic manner (151), potentially revealing biomarkers driving the proteome changes.

Additionally, several types of extracellular RNA originating from the brain can be transported, *via* extracellular vesicles, to the CSF and serum (152, 153). Hence, RNA-seq may be performed on CSF to reveal brain-derived mRNAs and microRNAs. Several differentially abundant microRNAs, long noncoding RNAs, as well as mRNAs have been found through liquid biopsies, for PD and AD (154, 155).

## Transcriptome-Based Applications in Clinical Practice

To our knowledge, no RNA-based biomarkers are currently used as a biomarker for diagnostics within the dementia field. However, the recent advances in detecting extracellular RNA described above suggest that RNA biomarkers may be feasible eventually.

While most neurodegenerative diseases are characterized by abnormal protein aggregates, it is important to note that the transcriptome to generate proteins can also be targeted to treat patients. By blocking the transcripts of mRNA *via* antisense oligonucleotides, it is possible to decrease the protein monomer concentrations of several proteins associated with amyloid fibril formation within the dementia pathologies. In mouse models, such therapies have been shown effective, and for several disease, (pre)clinical trials with antisense oligonucleotides are currently ongoing (156). Antisense oligonucleotides have been designed to lower expression of C9orf in models for ALS and FTD (157), targeting CAG repeats in the gene HTT in Huntington's disease (158, 159) and blocking CGG repeats in the FMR1 gene associated with FXTAS (160).

## Concluding Remarks

During recent years, we have seen enormous developments in biofluid-based biomarkers for neurodegenerative dementias. There are now clinically validated and approved CSF tests for AD pathologies and neurodegeneration (161), as well as promising developments in novel biomarkers for α-synuclein and TDP-43 pathologies (8, 133). We have also seen many of the AD and neurodegeneration biomarkers established as validated blood tests, for which appropriate use recommendations have been published (162). Although we need more biomarkers, it may be important to consider the underlying reasons for some of the successes that have been achieved.

The first and most obvious reason is technological improvement. Regular ELISA has become more sensitive, for example, through the incorporation of single molecule counting aspects using Single Molecule Counting or Simoa technologies. The mass spectrometers today are 10 to 100-fold more sensitive compared with only 10 years ago. For all technologies, automation has improved the analytical precision and sample throughput.

Another reason is that the cohorts in which novel biomarkers are discovered are much more deeply phenotyped today. Patients with clinical AD are made sure to be Aβ-positive, for example, by amyloid positron emission tomography or CSF Aβ42/40 ratio, before they are allowed into the AD group. And cognitively normal individuals are only included in the control group if they are amyloid-negative on positron emission tomography or CSF to ensure that they do not have preclinical AD. This leads to a considerable reduction in noise, which makes biomarker discovery and validation easier. Of note, such contrasts between disease and controls are relevant for initial discovery studies, while depending on the clinical questions, in later stages of development, a larger variation of comparison groups need to be included in the studies (113).

Finally, in targeted biomarker discovery projects, it has become more common to characterize the biomarker aspects of the pathology or pathophysiological process of interest in much greater molecular detail. For example, we now know that tau in brain tissue often is full-length. However, in biofluids, tau is mainly present as N-terminal fragments. This is likely an active enzymatic process happening in the neurons prior to or during secretion. Hence, targeting N-terminal tau is easier in biofluids than targeting full-length or C-terminal tau. However, there are also emerging data that C-terminal tau, measured using very sensitive assays, may be more directly reflective of what is going on in the brain tissue than N-terminal tau. This detailed molecular understanding of selective biomarker targets has facilitated biomarker development, validation, as well as interpretation.

**Fig. 2:**
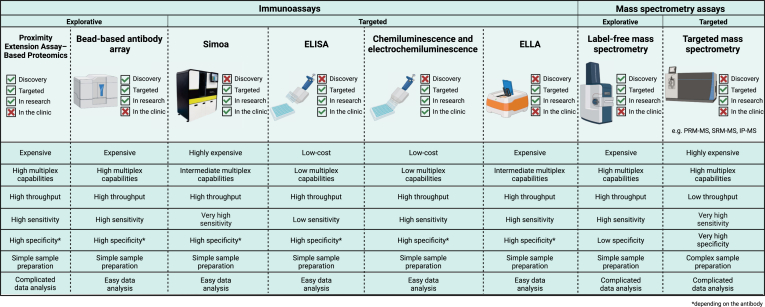
**Comparison of the different immunoassay platforms and mass spectrometry platforms discussed in this lexicon paper.** The qualifications are based on the information presented in the review as well as on expert opinion of the authors.

Improved methods for omics work with higher throughput has facilitated the recent paradigm shift from so-called “triangular” to “rectangular” study design in biomarker discovery projects (19, 50). In the classic triangular design, a small number of selected samples are characterized with extensive workflows and selected differentially expressed candidates are then assessed in a larger number of samples using targeted methods. In contrast, the rectangular strategy relies on multiple studies using the same workflow, moving the discovery to the population-wide setting to discern pathological from study-specific effects. Another important aspect to consider is that there is no single perfect biomarker discovery or validation method; all have their pros and cons and they can have complementary value (Fig. 2). The methods measure “sub-omes” and can hence be used together to increase coverage. Biomarker discoverers may want to combine them in an integrative approach that eventually may lead to network-based biomarker tools for precision medicine applications across neurodegenerative diseases (163). To achieve this, collaboration across laboratory disciplines and subdisciplines, in close interaction with clinical specialists and imaging experts, will be key.

## Conflicts of interest

S. D. is an employee of ADx NeuroSciences, Gent, Belgium. S. C. is an employee of National Measurement Laboratory at LGC, London, UK.

C. E. T. has a collaboration contract with ADx Neurosciences, Quanterix and Eli Lilly, performed contract research or received grants from AC-Immune, Axon Neurosciences, BioConnect, Biogen, Brainstorm Therapeutics, Celgene, EIP Pharma, Eisai, PeopleBio, Quanterix, Roche, Toyama, Vivoryon. She serves on editorial boards of Medidact Neurologie/Springer, Alzheimer Research and Therapy, Neurology: Neuroimmunology & Neuroinflammation, and is editor of a Neuromethods book Springer.

E. V. is a co-founder of ADx NeuroSciences.

H. Z. has served at scientific advisory boards and/or as a consultant for Abbvie, Acumen, Alector, Alzinova, ALZPath, Annexon, Apellis, Artery Therapeutics, AZTherapies, CogRx, Denali, Eisai, NervGen, Novo Nordisk, OptoCeutics, Passage Bio, Pinteon Therapeutics, Prothena, Red Abbey Labs, reMYND, Roche, Samumed, Siemens Healthineers, Triplet Therapeutics, and Wave, has given lectures in symposia sponsored by Cellectricon, Fujirebio, AlzeCure, Biogen, and Roche, and is a co-founder of Brain Biomarker Solutions in Gothenburg AB (BBS), which is a part of the GU Ventures Incubator Program (outside submitted work).

All other authors declare that they have no conflicts of interest with the contents of this article.
